# Dynamics of Bacterial Community and Fermentation Quality during Ensiling of Wilted and Unwilted *Moringa oleifera* Leaf Silage with or without Lactic Acid Bacterial Inoculants

**DOI:** 10.1128/mSphere.00341-19

**Published:** 2019-08-07

**Authors:** Yi Wang, Liwen He, Yaqi Xing, Yanting Zheng, Wei Zhou, Ruiqi Pian, Fuyu Yang, Xiaoyang Chen, Qing Zhang

**Affiliations:** a College of Forestry and Landscape Architecture, Guangdong Province Research Center of Woody Forage Engineering Technology, Guangdong Research and Development Centre of Modern Agriculture (Woody forage) Industrial Technology, Guangdong Key Laboratory for Innovative Development and Utilization of Forest Plant Germplasm, State Key Laboratory for Conservation and Utilization of Subtropical Agro-bioresources, Integrative Microbiology Research Centre, South China Agricultural University, Guangzhou, China; b College of Animal Science and Technology, China Agricultural University, Beijing, China; University of California, Davis

**Keywords:** *Moringa oleifera*, fermentation quality, inoculants, microbial community, wilting

## Abstract

To investigate the effects of wilting and lactic acid bacterial inoculants on the fermentation quality and bacterial community of Moringa oleifera leaf silage, fresh and wilted M. oleifera leaves were ensiled with or without Lactobacillus farciminis LF or Lactococcus lactis LL for 1, 7, 14, 30, and 60 days. The results showed that wilting, inoculants, and their interaction exerted significant (*P < *0.05) effects on the fermentation characteristics covering dry matter loss, pH value, lactic acid bacterial number, the ratio of lactic acid to acetic acid, and the relative abundances of bacteria, like for species of Lactobacillus, Lactococcus, Pediococcus, Enterococcus, Leuconostoc, and Enterobacter. Both LF and LL improved the fermentation quality of wilted and unwilted *M. oleifera* leaf silage by accelerating lactic acid production and pH decline, decreasing dry matter loss, and inhibiting yeast and coliform bacterial growth through the whole fermentation process. During ensiling, the abundances of *Lactococcus*, *Enterococcus,* and *Leuconostoc* spp. increased from day 1 to day 7 and then declined sharply from day 7 to day 14. Members of these genera and *Enterobacter* were inhibited, whereas *Lactobacillus* spp. were enhanced by these two lactic acid bacterial inoculants. The relative abundances of *Enterobacter*, *Enterococcus,* and *Pediococcus* spp. in inoculated silages were relatively low during the whole ensiling process. A lower abundance of *Enterobacter* spp. was observed in wilted silages than in unwilted silages. In summary, wilting and lactic acid bacterial inoculants had an influence on bacterial community and the fermentation process; LF and LL improved the fermentation quality of wilted and unwilted *M. oleifera* leaf silage.

**IMPORTANCE** *Moringa oleifera* leaf is a high-quality feed source for livestock and is increasingly used all over the world. Ensiling might be an effective method for preservation of the leaves. In the practice of silage making, lactic acid bacterial inoculants and wilting are commonly used to improve nutrition preservation. Monitoring the changes in a bacterial community during fermentation gives an insight into understanding and improving the ensiling process. Our results suggest that wilting and lactic acid bacterial inoculants had an influence on the bacterial community and fermentation process of *M. oleifera* leaf silage. Wilting showed positive effects on silage fermentation by decreasing the abundance of *Enterobacter* spp., while LF and LL improved the fermentation quality by inhibiting *Enterobacter* spp. and enhancing *Lactobacillus* spp. Both LF and LL accelerated the ensiling process from cocci (like *Lactococcus*, *Enterococcus,* and *Leuconostoc* spp.) to lactobacilli.

## Abstract (precis)

*Moringa oleifera* leaf is a high-quality feed source for livestock and is increasingly used all over the world. Ensiling might be an effective method for preservation of the leaves. In the practice of silage making, lactic acid bacterial inoculants and wilting are commonly used to improve nutrition preservation. Monitoring the changes in a bacterial community during fermentation gives an insight into understanding and improving the ensiling process. Our results suggest that wilting and lactic acid bacterial inoculants had an influence on the bacterial community and fermentation process of *M. oleifera* leaf silage. Wilting showed positive effects on silage fermentation by decreasing the abundance of *Enterobacter* spp., while LF and LL improved the fermentation quality by inhibiting *Enterobacter* spp. and enhancing *Lactobacillus* spp. Both LF and LL accelerated the ensiling process from cocci (like *Lactococcus*, *Enterococcus,* and *Leuconostoc* spp.) to lactobacilli.

## INTRODUCTION

Moringa oleifera Lam., originating in the Himalayas, is now widely cultivated for food, feed, and folk medicinal uses in many parts of tropical and subtropical regions of the world (1). It not only can adapt to all types of soils and humid, hot, and dry tropical conditions, but it also yields a large amount of fresh biomass, ranging from 43 to 115 tons per hectare annually (2). The *Moringa oleifera* tree is considered a miracle tree due to its potential uses in meeting human nutritional needs and in health promotion (3). *M*. *oleifera* leaves, an excellent source of protein and vitamins, provide a nutritious leaf vegetable in many developing countries where inadequate nourishment is of major concern. The leaves also contain abundant phytonutrients, like carotenoids, tocopherols, and ascorbic acid, which may scavenge free radicals and have immunosuppressive effects (4). The richness in proteins, essential amino acids, and minerals, along with its satisfactory fiber composition, have turned the leaves a potential high-quality feed source for livestock. Many studies found that providing *M. oleifera* for dairy cattle could improve feed utilization and milk production compared with traditional diets (2, 5, 6). In addition, replacing soybean meal with *M. oleifera* leaves could reduce CH_4_ production, thus mitigating greenhouse gas emissions (7).

Ensiling is a global practice to preserve the moist forage crop, especially in the rainy season, when drying forage is difficult (8). During ensiling process, lactic acid bacteria convert water-soluble carbohydrates into organic acids, mainly lactic acid. As a result of this acidification, undesirable microorganisms are inhibited and the silage is preserved. During the fermentation process, competition takes place between lactic acid bacteria and undesirable microorganisms. Therefore, the fermentation quality always depends on the result of the competition. However, to our knowledge, limited information is available on the characteristics of microbial community in *M. oleifera* leaf silage. Though our previous study reported on the microbial community composition and fermentation quality of *M. oleifera* leaf silage (9), the dynamic changes in bacterial and chemical compositions during the whole ensiling process have not been investigated.

It is known that cocci (such as *Leuconostoc*, *Pediococcus*, *Lactococcus,* and *Enterococcus* spp.) initiate lactic fermentation at the early stage of the ensiling process, while rod-shaped lactic acid bacteria (*Lactobacillus* spp.) dominate at the later stage (10). Furthermore, cocci and rod-shaped lactic acid bacteria are always used as silage inoculants to improve fermentation quality. However, it is not clear yet how the successive changes take place in silage and how these two kinds of lactic acid bacteria affect the fermentation process. On the other hand, wilting and adding lactic acid bacterial inoculants are conventional techniques to improve fermentation quality of silage. The application of these two techniques might have a great influence on bacterial community during the fermentation process.

Therefore, in the present study, we ensiled the fresh and wilted *M. oleifera* leaves with two lactic acid bacterial strains (Lactobacillus farciminis LF and Lactococcus lactis LL, previously isolated and selected from *M. oleifera* leaf silage). The fermentation quality and bacterial diversity of the silages were determined after 1, 7, 14, 30, and 60 days of ensiling.

## RESULTS AND DISCUSSION

### Characteristics of the raw material.

The chemical composition and microbial population of unwilted and wilted *M. oleifera* leaves prior to ensiling are shown in Table 1. Their dry matter (DM) contents were 245 and 448 g/kg, respectively. The crude protein content (about 260 g/kg [DM]) was comparable with the data reported by Zheng et al. (11) but far higher than the results from our previous study (9). The relatively high crude protein content and low fiber contents (257 and 287, and 176 and 190 g/kg [DM] for neutral detergent fiber and acid detergent fiber, respectively) suggested that *M. oleifera* leaves could potentially be used as a kind of quality protein fodder for livestock. The chemical composition of silage material, especially the content of water-soluble carbohydrates, plays a critical role in assessing fermentation quality. The water-soluble carbohydrate content (99.0 and 95.5 g/kg [DM] in unwilted and wilted *M. oleifera* leaves, respectively) was higher than the threshold (60 to 70 g/kg DM) for well-preserved silage (12) and was comparable with results from our previous study (100.7 g/kg [9]). The relatively high water-soluble carbohydrate content might explain the good quality of *M. oleifera* leaf silage. Generally, >5.0 log CFU/g (fresh matter [FM]) lactic acid bacteria at ensiling is necessary for well-preserved silage (13). In the present study, the lactic acid bacterial counts were 5.36 and 5.09 log CFU/g (FM) in unwilted and wilted *M. oleifera* leaves, respectively. The relative high lactic acid bacterium counts might be helpful for the fermentation process.

**TABLE 1:** Chemical composition and microbial population in unwilted and wilted *M. oleifera* leaves prior to ensiling

Measurement^*a*^	Value ± SD by silage leaf type (*n* = 3)	
	Unwilted	Wilted
Dry matter (g/kg [FM])	245 ± 1.8	448 ± 5.8
Crude protein (g/kg [DM])	264 ± 11.4	260 ± 0.6
Neutral detergent fiber (g/kg [DM])	257 ± 6.0	287 ± 6.8
Acid detergent fiber (g/kg [DM])	176 ± 8.7	190 ± 11.3
Water-soluble carbohydrate (g/kg [DM])	99.0 ± 8.0	95.5 ± 7.4
Lactic acid bacteria (log_10_ CFU/g [FM])	5.36 ± 0.13	5.09 ± 0.19
Yeasts (log_10_ CFU/g [FM])	<2.00	<2.00
Molds (log_10_ CFU/g [FM])	3.71 ± 0.18	2.30 ± 0.43
Coliform bacteria (log_10_ CFU/g [FM])	5.78 ± 0.18	5.33 ± 0.28

a FM, fresh matter; DM, dry matter.

### Fermentation quality of *M. oleifera* leaf silage during ensiling.

**FIG 1:**
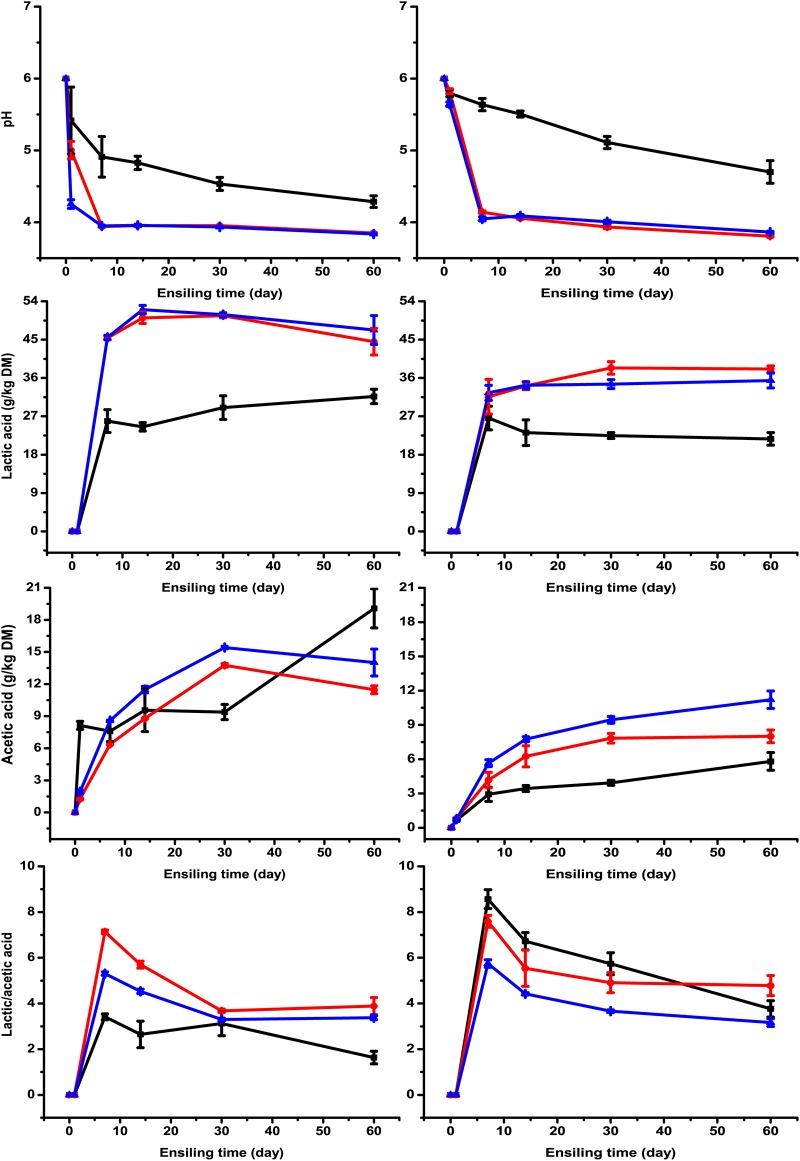
pH and contents of lactic acid, acetic acid, and lactic/acetic acid in unwilted (left) and wilted (right) *M. oleifera* leaves ensiled without (black lines with squares) or with Lactobacillus farciminis LF (red lines with circles) or Lactococcus lactis LL (blue lines with triangles) after 1, 7, 14, 30, and 60 days of ensiling.

The dynamic changes in pH and organic acid contents during fermentation are shown in Fig. 1. Overall, the pH value and lactic acid and acetic acid contents in wilted and unwilted silage showed a similar pattern during the ensiling process, where the pH value decreased while the concentrations of the two acids increased during fermentation. The pH value was decreased and lactic acid was increased by the two lactic acid bacterial strains, LF and LL, compared to the control. Factorial analysis revealed that wilting, inoculants, and their interaction had significant effects (*P < *0.01) on pH value, the contents of lactic acid and acetic acid, and the ratio of lactic acid to acetic acid (Table 2). Propionic acid and butyric acid were not detected during the whole ensiling process.

**TABLE 2:** Significant analysis of wilting, ensiling time, inoculants, and their interactions on fermentation characteristics and the relative abundances of main genera of *M. oleifera* leaf silage

Characteristic or abundance	*P* value^*a*^						
	W	E	I	W × E	W × I	E × I	W × E × I
Characteristic							
Dry matter loss	<0.01	<0.01	<0.01	<0.01	<0.01	<0.01	<0.01
pH	<0.01	<0.01	<0.01	<0.01	<0.01	<0.01	<0.01
Lactic acid bacterial content	<0.01	<0.01	<0.01	<0.01	<0.01	<0.01	<0.01
Coliform bacterial content	NS	<0.01	<0.01	NS	NS	<0.01	NS
Yeast content	<0.01	<0.01	<0.01	NS	NS	<0.01	
Lactic acid content	<0.01	<0.01	<0.01	NS	<0.01	<0.01	<0.01
Acetic acid content	<0.01	<0.01	<0.01	<0.01	<0.01	<0.01	<0.01
Lactic/acetic acid content	<0.01	<0.01	<0.01	<0.01	<0.01	<0.01	<0.01
Abundance by genus							
*Lactobacillus*	<0.01	<0.01	<0.01	<0.01	<0.01	<0.01	<0.01
*Lactococcus*	<0.01	<0.01	<0.01	<0.01	<0.01	<0.01	<0.01
*Pediococcus*	<0.01	<0.01	<0.01	<0.01	<0.01	<0.01	<0.01
*Enterococcus*	NS	<0.05	<0.01	<0.01	NS	<0.01	<0.01
*Leuconostoc*	<0.01	<0.01	<0.01	<0.01	<0.01	<0.01	<0.01
*Enterobacter*	<0.01	<0.01	<0.01	<0.01	<0.01	<0.01	<0.01
*Pseudomonas*	<0.01	<0.01	NS	<0.01	NS	<0.01	NS
*Streptococcus*	NS	NS	NS	NS	NS	NS	NS
*Acinetobacter*	NS	<0.01	NS	NS	<0.05	NS	<0.01
*Xanthomonas*	<0.01	<0.01	<0.01	<0.01	<0.01	<0.01	<0.01

a W, wilting; E, ensiling time; I, inoculants; NS, not significant.

Silage pH is an important indicator for assessing fermentation quality, especially for high-moisture silages. The decrease of pH value mainly occurred in the first 7 days of ensiling, whereas no further decline was observed with prolonged ensilage time. This is in accordance with the result reported by Ni et al. (14). Comparison of the two uninoculated silages (wilted and unwilted) indicated that the pH decreased less extensively in wilted silage than in unwilted silage. This might be because the low moisture content limited the activity of lactic acid-producing bacteria. The final pH values in the two wilted and unwilted uninoculated groups were 4.28 and 4.70, respectively, which might indicate that ensiling is a feasible way for *M. oleifera* leaf preservation. Lactic acid bacterial inoculants are commonly used in silage making in order to obtain favorable fermentation by accelerating pH decline and stimulating lactic acid accumulation. In the present study, benefits of lactic acid bacterial inoculants were found when silage was treated with LF and LL. Rapid pH decline and lactic acid accumulation were seen in the early stage of ensiling (Fig. 1). The pH value decreased more extensively by the two inoculants in unwilted silage than in wilted silage after 3 days of ensiling. This indicates that moisture content has an impact on the efficiency of inoculants during ensiling. A pH of <4.2 is always used as a benchmark for well-fermented silage; otherwise, putrefaction of silage will appear in high-moisture silages (15). The pH of all inoculated silage samples was below 4.2 after 7 days of ensiling. The fast decline in pH in the two lactic acid bacterial strain-treated silages could ensure good preservation of *M. oleifera* leaves and also explain the absence of butyric acid and coliform bacteria (<2.00 log CFU/g [FM]). As expected, lactic acid content was increased by LF and LL in both wilted and unwilted silages. This indicates that adding lactic acid bacterial inoculants is helpful in *M. oleifera* leaf silage making.

The result that prolonged storage enhanced acetic acid production agreed well with results from previous studies (16, 17). Li and Nishino (17) speculated that the increase in acetic acid should be attributed to the decrease in Enterococcus sulfureus and the increase in Lactobacillus plantarum. Lactobacillus plantarum is considered a facultatively heterofermentative species which could produce acetic acid during ensiling. The ratio of lactic acid to acetic acid is an indicator of the extent of homofermentation in relation to heterofermentation during ensiling. In this study, the ratio decreased during the ensiling process. This might be attributed to the increase in acetic acid. The decline of the ratio together with the accumulation of acetic acid might be caused by the enhanced activity of heterofermentative lactic acid bacteria.

**FIG 2:**
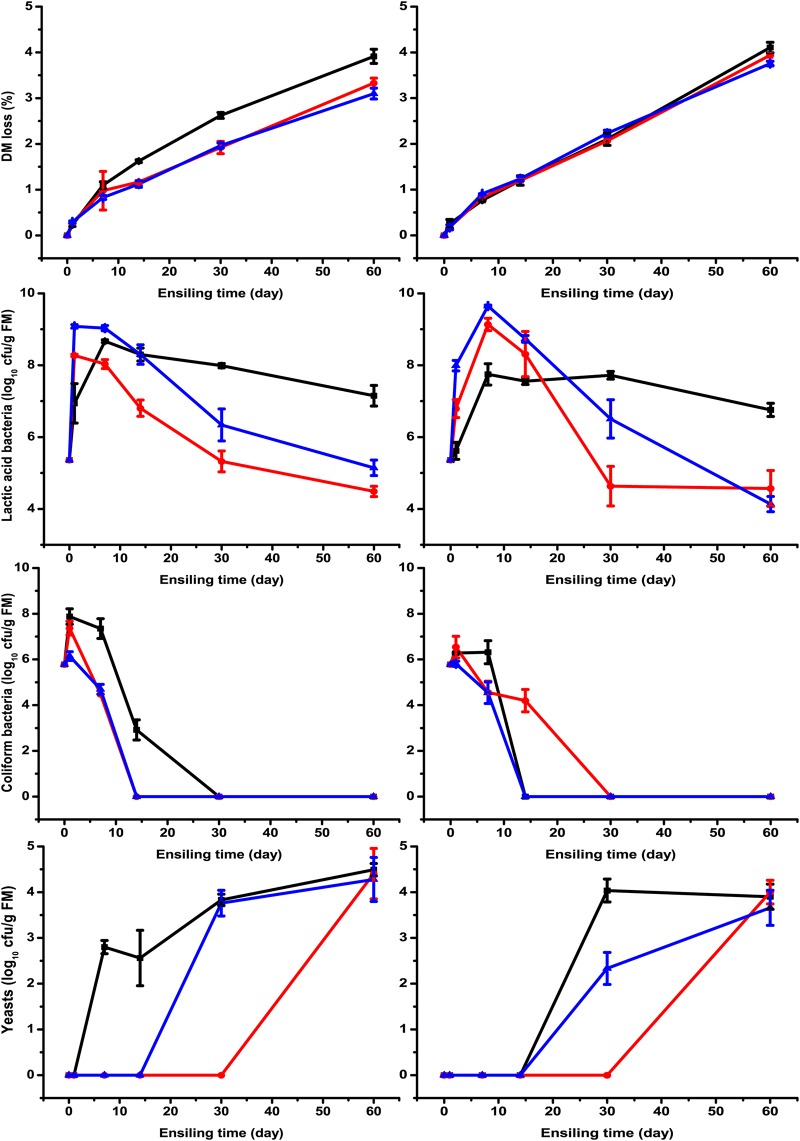
DM loss and counts of lactic acid bacteria, coliform bacteria, and yeasts in unwilted (left) and wilted (right) *M. oleifera* leaves ensiled without (black lines with squares) or with Lactobacillus farciminis LF (red lines with circles) or Lactococcus lactis LL (blue lines with triangles) after 1, 7, 14, 30, and 60 days of ensiling.

As shown in Fig. 2, more lactic acid bacteria were detected in LF- and LL-treated silages than in the untreated control after 1 day of ensiling. This means that exogenous addition of lactic acid bacterial inoculants is effective and is consistent with the reduction in pH and lactic acid accumulation at the early stage of ensiling. Wilting, inoculants, and their interaction had significant effects (*P < *0.01) on lactic acid bacterial count (Table 2). The interaction indicates that the impact of inoculants on lactic acid bacterial count would be different with different moisture contents. In the present study, the lactic acid bacterial count was lower in wilted silage than in unwilted silage in a comparison of the two uninoculated groups. However, the lactic acid bacterial count was higher in wilted silage after treatment with inoculants. Similarly, our previous study also found the interaction between inoculants and wilting, where inoculants were more effective in increasing lactic acid bacterial count in wilted silage than in unwilted silage (9). When storage was extended, the number of lactic acid bacteria decreased, where the decrease was more apparent in LF- and LL-treated groups. The rapid acidification and antagonistic activity might suppress the activity of lactic acid bacteria. Similarly, Li and Nishino (18) found that the lactic acid bacterial number in wilted Italian ryegrass silage was higher in the Lactobacillus rhamnosus-treated group than in the control at day 14 of ensiling, while the opposite result was observed at day 56. The coliform bacterial count was below 2.00 log CFU/g (FM) in all silage samples after 30 days of ensiling and was lower in wilted silage than in unwilted silage. This might be because the relatively low pH value and moisture content limited the growth of coliform bacteria.

Ni et al. (14) found that the number of yeasts in soybean silage increased (7 to 8 log CFU/g [FM]) at the beginning stage of ensiling and then decreased (about 5 log CFU/g [FM]) as a result of pH reduction. However, in the present study, the yeasts in all treatments increased during the whole ensiling process, though the final count was lower than 5 log CFU/g FM. This might be because many yeasts strains are capable of growing at pH 3.5. Muck (19) considered that when enough sugars were remained in silages after the lactic acid bacteria were inhibited by low pH, yeasts might develop. Overall, it is difficult to explain the increase in yeasts, and further research into the change of fungal community in *M. oleifera* leaf silage is needed. In our study, dry matter loss of silages increased during fermentation. This might be because of the activities of coliform bacteria and yeasts. During ensiling, coliform bacteria may compete with the lactic acid bacteria for nutrients and produce silo gas, succinic acid, and 2,3-butanediol (19), and then the dry matter loss occurs. The metabolism of yeasts, which utilizes soluble carbohydrates and produces alcohol, also results in dry matter loss (20).

### Microbial community of *M. oleifera* leaf silage during ensiling.

**FIG 3:**
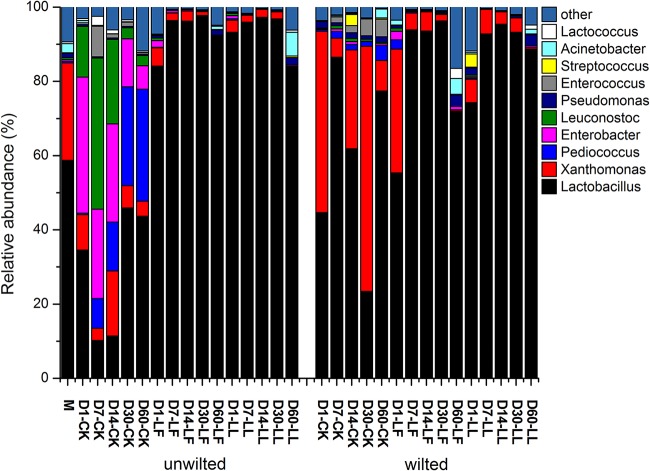
Bacterial communities and relative abundances by genus for unwilted and wilted *M. oleifera* leaves ensiled without or with Lactobacillus farciminis LF or Lactococcus lactis LL after 1, 7, 14, 30, and 60 days of ensiling. M, material; CK, control.

**FIG 4:**
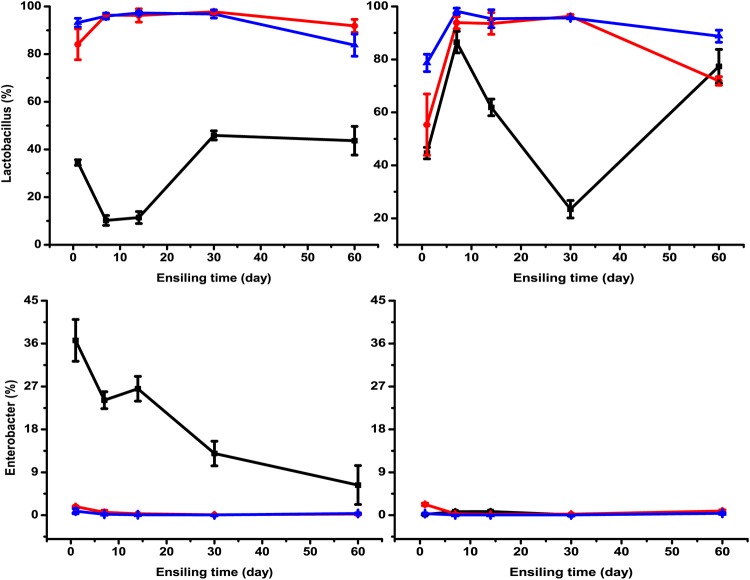
The relative abundances of *Lactobacillus* and *Enterobacter* spp. in unwilted and wilted *M. oleifera* leaves ensiled without (black lines with squares) or with Lactobacillus farciminis LF (red lines with circles or Lactococcus lactis LL (blue lines with triangles) after 1, 7, 14, 30, and 60 days of ensiling.

**FIG 5:**
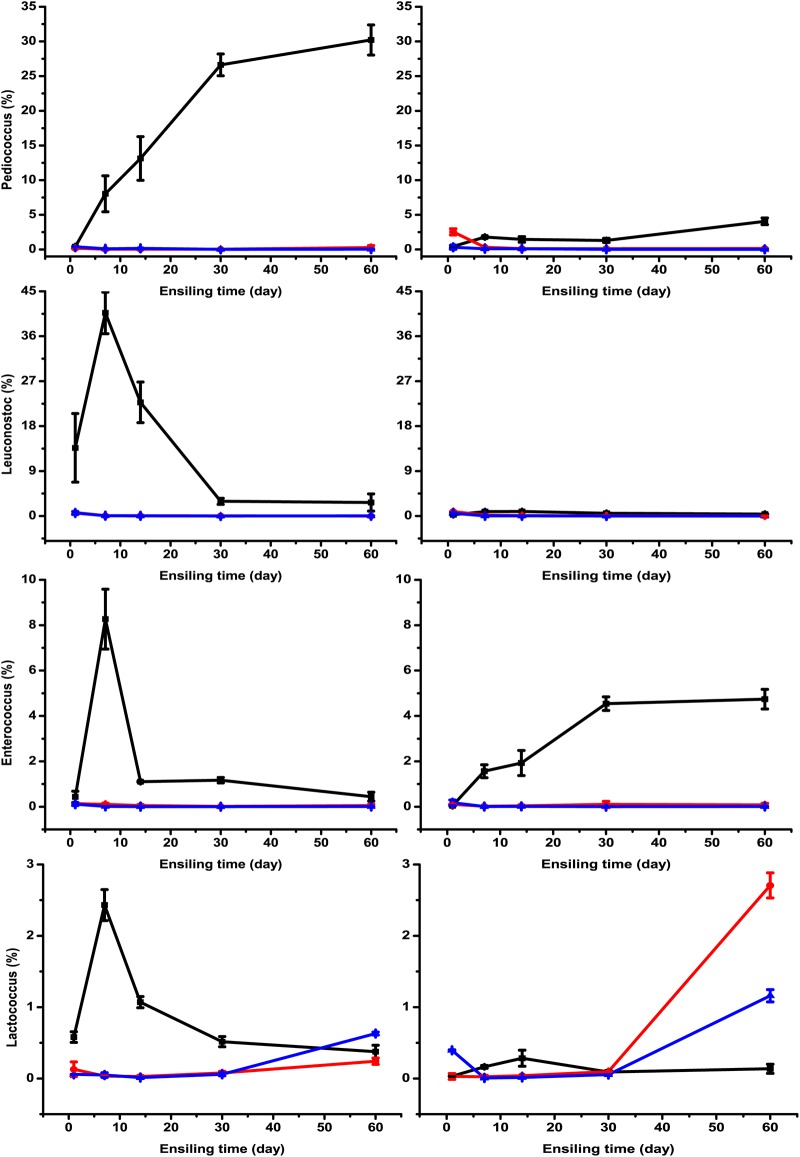
The relative abundances of *Pediococcus*, *Leuconostoc*, *Enterococcus,* and *Lactococcus* spp. in unwilted and wilted *M. oleifera* leaves ensiled without (black lines with squares) or with Lactobacillus farciminis LF (red lines with circles) or Lactococcus lactis LL (blue lines with triangles) after 1, 7, 14, 30, and 60 days of ensiling.

The microorganisms in silage play a critical role in the fermentation process. Monitoring the changes in the bacterial community during fermentation gives an insight into understanding and improving the ensiling process (21). The main bacterial communities in *M. oleifera* leaf silage are shown in Fig. 3. *Lactobacillus* was the dominant genus in LF- and LL-treated silages (55% to 98%), which might explain their relatively high fermentation quality. However, this is inconsistent with Wang et al. (9), who reported that Exiguobacterium spp. were dominant in *M. oleifera* leaf silage. This might be because bacterial species and population might vary according to climate and growth stage (22). The changes in microbial community in Rhodes grass silage were measured using denaturing gradient gel electrophoresis (DGGE) by Parvin and Nishino (23), where the band of Lactococcus lactis was strong after 15 days of ensiling and became fainter but did not disappear. Similarly, in the present study, *Lactococcus* spp. were most abundant at day 7 and declined subsequently in unwilted and uninoculated silages (Fig. 4). Likewise, Brusetti et al. (24) found that Lactococcus lactis subsp. lactis reached its highest level at day 6 using length heterogeneity PCR (LH-PCR) electropherograms. Xu et al. (21) also detected a significant increase and then reduction in the abundance of Lactococcus lactis in corn silage from day 0 to 10 using a high-throughput sequencing method. A similar pattern for *Leuconostoc* and *Enterococcus* spp. was observed during the ensiling process. Recently, Yang et al. (25) detected the bacterial community in alfalfa silage inoculated with or without Lactobacillus plantarum and found that *Leuconostoc* and *Enterococcus* spp. declined after 7 or 14 days of ensiling. This might be because *Enterococcus*, *Lactococcus,* and *Leuconostoc* spp. initiate silage fermentation and create a suitable environment for the development of lactobacilli, and then those cocci are replaced by more acid-tolerant lactobacilli like Lactobacillus plantarum and Lactobacillus brevis (10, 13). This might partially explain the change in *Lactobacillus* spp. in wilted *M. oleifera* leaf silage, which decreased from day 7 to day 30 and increased obviously from day 30 to day 60 (Fig. 5). A similar result was reported by Li and Nishino (17), who determined the bacterial community in wilted guinea grass silage using DGGE and found that the band representing Lactobacillus garvieae was far fainter at day 28 than at day 14 and day 56. Furthermore, Yang et al. (25) also reported that the relative abundances of *Leuconostoc* and *Enterococcus* spp. were decreased by Lactobacillus plantarum inoculation. In the present study, ensiling time, lactic acid bacterial inoculants, and their interaction had significant effects (*P < *0.01) on the relative abundances of *Lactobacillus*, *Lactococcus*, *Pediococcus*, *Enterococcus*, *Leuconostoc,* and *Enterobacter* spp. (Table 2). For example, the relative abundance of *Pediococcus* spp. increased with prolonged ensilage time, but it remained almost the same after being treated with the two inoculants. The interaction of ensiling time and lactic acid bacterial inoculants indicates that the effects of the inoculants on microbial community change with the ensiling time, and the inoculants can alter the ensiling process. Similar effects also had been reported by Yang et al. (25). *Lactobacillus* spp. were more abundant in LF- or LL-treated silages than in the control. The relative abundances of *Pediococcus*, *Leuconostoc,* and *Enterococcus* spp. in LF- and LL-treated silages were low during the whole ensiling process. Similar results were reported by Eikmeyer et al. (26) and Romero et al. (27), who found that Lactobacillus buchneri inoculation increased the abundance of *Lactobacillus* spp. and decreased the abundances of *Pediococcus*, *Leuconostoc,* and *Lactococcus* spp. This might be because the rapid decline in pH that resulted from the inoculation decreased the abundance of these genera. All the above-mentioned results indicate that the lactic acid bacterial inoculants might benefit silage by accelerating fermentation to a stage where *Lactobacillus* spp. were dominant. Species of *Pediococcus* are usually used as silage inoculants due to their acid tolerance and lactic acid production. Furthermore, *Pediococcus* spp. grow rapidly at the early stage of ensiling when the pH is between 5 and 6.5 (28, 29). Therefore, *Pediococcus* spp. could promote pH reduction during the early stage of fermentation. The increase in *Pediococcus* spp. with ensiling time in this study was in accordance with the results from Stevenson et al. (30), who detected several species of lactic acid bacteria using real-time PCR (RT-PCR). Likewise, Zheng et al. (31) determined bacterial community in alfalfa silage using high-throughput sequencing and found that *Pediococcus* spp. increased with ensiling time.

During ensiling, the presence of *Enterobacter* spp. is undesirable, as they may compete with the lactic acid bacteria for nutrients, produce ammonia-N, and cause nutrition loss. In the present study, the relative abundance of *Enterobacter* spp. decreased from 37% to 6% after fermentation in unwilted and uninoculated silage (Fig. 3 and 5). This is consistent with the results from Parvin et al. (32), who reported an evolution in the bacterial community from *Enterobacter* spp. to *Lactobacillus* spp. and *Lactococcus* spp. after ensiling of whole-corn silage. The restriction in fermentation of low moisture was reflected by the lower abundance of *Enterobacter* spp. in wilted silages. The relative abundance of *Enterobacter* spp. in LF- and LL-treated silages was low during the whole ensiling process. This might be because the fast decline of pH caused by the addition of inoculants inhibited the activities of *Enterobacter* spp. Similarly, Zheng et al. (31) also found that the relative abundance of *Enterobacter* spp. declined with increased ensiling time and decreased after treatment with Lactobacillus plantarum.

Pseudomonas spp. might be undesirable in silage due to the possibility of biogenic amine production (22). Acinetobacter species are aerobic bacteria and can be found in different environments. Fuhs and Chen (33) found that some Acinetobacter species can utilize acetate as a substrate and survive in an anaerobic environment. The utilization of acetic acid by Acinetobacter spp. in an anaerobic environment requires energy from carbohydrate degradation; thus, silage DM loss increases during ensiling. The good news is that the two genera are not abundant in *M. oleifera* leaf silage (3% and 6% at maximum, respectively; Fig. 3). On the other hand, Acinetobacter spp. might be concerned with the aerobic stability of silage. Liu et al. (34) investigated the bacterial community in barley silage during the fermentation process and aerobic exposure phase and found that species of Acinetobacter proliferated rapidly and that Acinetobacter became the dominant genus after 7 days of exposure to air. In the present study, Acinetobacter spp. were more commonly observed in silages fermented for 60 days. Therefore, studies on the aerobic stability of *M. oleifera* leaf silage and their relationship with Acinetobacter spp. might be conducted in the future. It should be noted that Xanthomonas is one of the most abundant groups of plant-pathogenic bacteria and can cause a variety of diseases in many crops (35). This genus was also detected by Minh et al. in rice straw silage (36). It declined in LF and LL treatments in the present study. This indicates that the addition of the two strains may control this kind of pathogenic bacteria during ensiling.

### Conclusions.

This study found that lactic acid bacteria inoculants, LF and LL, improved the fermentation quality of wilted and unwilted *M. oleifera* leaf silage. Lactic acid accumulation and pH decline were accelerated and dry matter loss was decelerated by the two lactic acid bacterial inoculants during the whole fermentation process. The abundances of *Lactococcus*, *Enterococcus,* and *Leuconostoc* spp. increased from day 1 to day 7 and then declined sharply from day 7 to day 14. These genera and *Enterobacter* spp. were inhibited, whereas *Lactobacillus* spp. were enhanced by the two lactic acid bacterial inoculants. *Enterobacter* spp. were observed at lower abundance in wilted silages than in unwilted silages. In summary, wilting and lactic acid bacterial inoculants had positive effects on the fermentation process of *M. oleifera* leaf silage.

## MATERIALS AND METHODS

Overall, the experiment was conducted as per the following procedures. *Moringa oleifera* leaves were manually collected and were partially wilted for 5 h. The unwilted and wilted leaves were chopped and ensiled without (the control) or with two lactic acid bacterial strains, Lactobacillus farciminis LF and Lactococcus lactis LL. For each treatment, 15 minisilos were prepared, and three minisilos were opened after 1, 7, 14, 30, and 60 days of ensiling. Finally, raw material and silage samples were analyzed for fermentation quality and bacterial community.

### Raw material and silage preparation.

*Moringa oleifera* leaves (3 months after previous harvest) were manually collected from the experimental field at the South China Agricultural University (Guangzhou, China) in June 2018. No herbicides or fertilizers were used during planting. The leaves without wilting and those wilted (in 28 to 30°C, 70% relative humidity, and windless conditions) for 5 h were chopped to 1 to 2 cm by a paper cutter. Two lactic acid bacterial strains, Lactobacillus farciminis LF (GenBank accession number MK524159) and Lactococcus lactis LL (GenBank accession number MK524164), were isolated from *M. oleifera* leaf silage due to their high growth rate and acid productivity. The lactic acid bacterium powder was prepared according to Zhang et al. (37). Accurately weighed lactic acid bacterium powder was mixed with 4 ml of sterile distilled water and sprayed onto the 200 g of chopped *M. oleifera* leaves to achieve a dose of 5 log CFU/g (fresh matter [FM]). The control was added with the same volume of distilled water. Then, about 200 g of *M. oleifera* leaves was immediately packed into plastic silo bags (20 cm height and 30 cm length; Dongguan Bojia Packaging Co. Ltd., Dongguan, China). Subsequently, a vacuum sealer (Lvye DZ280; Dongguan Yijian Packaging Machinery Co. Ltd.) was used to vacuum and seal these bags. In total, 90 bags (2 dry matter × 3 treatments × 5 ensiling periods × 3 repeats) were made and kept at ambient temperature (25 to 32°C). Three minisilos for each treatment were opened to determine fermentation quality and bacterial community after 1, 7, 14, 30, and 60 days of ensiling.

### Analysis of microbial population, organic acids, and chemical composition.

To determine the population of microorganisms, 20-g samples were immediately blended with 180 ml sterilized saline water (8.5 g/liter NaCl) and serially diluted from 10^−1^ to 10^−6^. The numbers of lactic acid bacteria, coliform bacteria, and yeasts and molds were incubated and counted using de Man-Rogosa-Sharpe (MRS) agar, Violet red bile agar, and Rose Bengal agar, respectively (9).

Twenty grams of each silage sample was mixed with 180 ml distilled water, stored at 4°C for 18 h, and then filtered. The pH of this filtrate was measured by a glass electrode pH meter (PHS-3C; INESA Scientific Instrument Co., Ltd., Shanghai, China). A high-performance liquid chromatography (HPLC) column (Shodex RSpak KC-811S-DVB gel C, 8.0 mm by 30 cm; Shimadzu, Tokyo, Japan) with an oven temperature of 50°C, mobile phase of 3 mmol/liter HClO_4_, flow rate of 1.0 ml/min, and injection volume of 5 μl, and the SPD-M10AVP detector was used to measure the concentrations of organic acids (lactic acid, acetic acid, propionic acid, and butyric acid) (37).

About 100 g *M. oleifera* leaf raw material and silage samples was dried at 65°C for 48 h to determine dry matter content. The dried material samples were ground to pass through a 1-mm screen using a laboratory knife mill (FW100; Taisite Instrument Co., Ltd., Tianjin, China). Crude protein was analyzed using a Kjeldahl nitrogen analyzer (Kjeltec 2300 autoanalyzer; Foss Analytical AB, Höganäs, Sweden) according to the methods of the Association of Official Analytical Chemists (38). According to the method of Van Soest et al. (39), an A220 fiber analyzer (Ankom Technology Corp., Macedon, NY, USA) was used to measure neutral detergent fiber and acid detergent fiber contents. The content of water-soluble carbohydrates was determined using the anthrone method (40).

### Microbial diversity analysis.

Samples (10 g) were mixed with 90 ml of sterile 0.85% NaCl solution with vigorous shaking at 120 rpm for 2 h. The mixture was filtered through four layers of cheesecloth, and the filtrate was centrifuged at 10,000 rpm for 10 min at 4°C. The deposit was resuspended in 1 ml of sterile 0.85% NaCl solution, and the microbial pellets were obtained by centrifugation at 12,000 rpm for 10 min at 4°C. To extract total DNA in raw material and silage samples (93 samples in total), the E.Z.N.A. stool DNA kit (Omega Bio-tek, Norcross, GA) was used, according to the manufacturer’s protocols. The PCRs were conducted in a 50-μl mixture (100 ng of template DNA, 5 μl of 2.5 mM dinucleoside triphosphates [dNTPs], 1.5 μl of each primer [5 μM], 1 μl of KOD polymerase, and 5 μl of 10× KOD buffer). According to Wang et al. (9), the 16S rRNA gene V3-V4 regions were amplified using primers 341F (CCTACGGGNGGCWGCAG) and 806R (GGACTACHVGGGTATCTAAT).

After purification and quantification, the PCR products were sequenced using an Illumina platform (Guangzhou Gene Denovo Co. Ltd., Guangzhou, China). The raw sequences were selected according to Wang et al. (9). Paired-end clean reads were merged as raw tags using FLASH (v 1.2.11), with a minimum overlap of 10 bp and mismatch error rates of 2%. Noisy sequence filtering and data processing were performed using QIIME (v 1.9.1). After deleting unqualified sequences, the valid sequences of all 93 samples summed 12,965,265, with an average length of 431 bp per sequence for bacteria. Clean tags were searched against the reference database (http://drive5.com/uchime/uchime_download.html) to perform reference-based chimera checking using the UCHIME algorithm (http://www.drive5.com/usearch/manual/uchime_algo.html). Chimeric sequences were removed, and the effective tags with 0.97 identities were clustered into operational taxonomic units (OTU) using the UPARSE pipeline. The analysis of taxonomy assignment of representative sequences was performed using Ribosomal Database Project (RDP) Classifier (version 2.2).

### Statistical analyses.

The fermentation characteristics and the relative abundances of main genera of *M. oleifera* leaf silage were analyzed as a 2 × 3 × 5 factorial design study with three replicates per treatment. The effects of wilting, lactic acid bacterial inoculants, and ensiling time were evaluated using two-way analysis of variance, with Duncan’s multiple-range tests. All statistical procedures were conducted using the SAS 9.3 software (SAS Institute, Inc., Cary, NC, USA). The data from high-throughput sequencing were analyzed using the OmicShare tools, a free online platform for data analysis (http://www.omicshare.com/tools).

### Data availability.

The 16S rRNA sequences of the isolates described in this report were deposited in GenBank under accession numbers MK524159 to MK524164. In addition, all raw sequencing reads have been deposited in the NCBI Sequence Read Archive (SRA) under accession number SRP186719.
